# Automated Regional Detection of Intervortex Venous Anastomosis on Ultra-Widefield Indocyanine Green Angiography Using a Two-Stage Deep Learning Framework

**DOI:** 10.3390/jcm15197709

**Published:** 2026-10-06

**Authors:** Areum Jeong, Min Sagong

**Affiliations:** 1 Department of Ophthalmology, College of Medicine, Yeungnam University, Daegu 42415, Republic of Korea; eyeareum@gmail.com; 2 Yeungnam Eye Center, Yeungnam University Hospital, Daegu 42415, Republic of Korea

**Keywords:** intervortex venous anastomosis, vortex vein, ultra-widefield imaging, indocyanine green angiography, deep learning, choroid

## Abstract

**Objectives:** This study aimed to develop and validate a deep learning–based system for automated detection of intervortex venous anastomosis (IVA) on ultra-widefield indocyanine green angiography (UWF-ICGA). **Methods**: A total of 183 UWF-ICGA images with an original resolution of 3900 × 3072 pixels were included. A two-stage deep learning framework was developed. In the first stage, an object detection model automatically localized four predefined quadrants within each UWF-ICGA image. In the second stage, each extracted quadrant was classified according to the presence or absence of IVA. Quadrants demonstrating definite venous connections between adjacent vortex vein drainage territories were defined as IVA-positive, whereas quadrants without identifiable intervortex venous connections were defined as IVA-negative. Brightness normalization was applied before analysis, and images were resized to 512 × 512 pixels using linear interpolation. The object detection model was trained using 155 images and tested using 28 images. The 183 original images generated 732 quadrant images, of which 622 were used for training and 110 for testing of the classification model. Model performance was evaluated using accuracy, precision, recall, F1 score, and confusion matrices. **Results**: The object detection model correctly localized all predefined quadrants in the test dataset, achieving an accuracy, precision, recall, and F1 score of 100.0%. All 112 manually labeled regions from 28 test images were correctly matched by the model. The IVA classification model achieved an accuracy of 93.63%, precision of 95.39%, recall of 91.46%, and F1 score of 93.38%. Among 110 test quadrants, all 69 IVA-negative quadrants were correctly classified, while 34 of 41 IVA-positive quadrants were correctly identified. Seven IVA-positive quadrants were misclassified as negative, whereas no IVA-negative quadrant was classified as positive. When IVA-positive was considered the positive class, the corresponding sensitivity and specificity were 82.9% and 100%, respectively. **Conclusions**: A two-stage deep learning system enabled automated detection of IVA on UWF-ICGA with high classification performance. Automated assessment of IVA may provide an objective and reproducible approach for evaluating choroidal venous remodeling and may serve as a platform for future quantitative investigations of vortex vein abnormalities.

## 1. Introduction

The choroidal circulation is primarily drained through the vortex vein system, which is organized into distinct venous drainage territories separated by horizontal and vertical watershed zones [1]. Under normal conditions, individual vortex vein systems largely drain their respective territories. However, alterations in choroidal venous outflow may result in dilation and remodeling of vortex veins, with the development of venous connections across conventional watershed boundaries.

Intervortex venous anastomosis (IVA) has emerged as an important imaging feature of choroidal venous remodeling. Previous studies using widefield and ultra-widefield indocyanine green angiography (ICGA) have demonstrated abnormal connections between vortex vein systems in eyes with pachychoroid spectrum diseases. Matsumoto et al. reported superior–inferior vortex vein anastomosis in more than 90% of eyes with central serous chorioretinopathy (CSC), pachychoroid neovasculopathy (PNV), and polypoidal choroidal vasculopathy (PCV), substantially more frequently than in healthy eyes [2]. These findings suggested that longstanding choroidal venous congestion may promote formation of collateral drainage pathways through intervortex venous anastomoses. Building on these observations, Spaide et al. proposed the concept of “venous overload choroidopathy,” providing a unifying framework in which increased choroidal venous pressure or impaired venous outflow leads to venous dilation, remodeling, and formation of intervortex venous anastomoses [3]. In this model, IVA may represent an adaptive response to chronic venous overload by providing alternative pathways for choroidal venous drainage. The framework provides a mechanistic explanation linking vortex vein abnormalities with the characteristic choroidal changes observed in CSC and related pachychoroid disorders [3].

The increasing recognition of IVA has therefore shifted attention from choroidal thickness alone toward the organization and remodeling of the choroidal venous circulation. Ultra-widefield ICGA (UWF-ICGA) is particularly well suited for this purpose because it allows simultaneous visualization of multiple vortex vein drainage territories, including peripheral portions of the venous system that may not be captured by conventional posterior-pole imaging. Widefield imaging has demonstrated that IVA may occur in multiple choroidal sectors and has provided further evidence that these connections represent a component of broader vortex vein remodeling [3,4,5,6]. Despite its potential clinical and pathophysiological relevance, evaluation of IVA on UWF-ICGA remains largely qualitative and depends on manual interpretation of complex choroidal vascular networks. Identification of anastomotic connections across multiple peripheral regions can be time-consuming and may be affected by image quality and examiner experience. Furthermore, the lack of a standardized method for detecting IVA limits reproducibility and may hinder large-scale investigation of choroidal venous remodeling.

Deep learning provides an opportunity to automate this process. Although artificial intelligence has been extensively applied to retinal image classification, segmentation, and lesion detection, automated assessment of the peripheral choroidal venous system has received relatively little attention. Because IVA represents a spatially distributed vascular feature, a regional approach may be particularly suitable for automated analysis of UWF-ICGA. Therefore, the purpose of this study was to develop and validate a two-stage deep learning framework for automated detection of IVA on UWF-ICGA. The proposed system first automatically localized four predefined regions of each UWF-ICGA image and subsequently classified each region according to the presence or absence of IVA.

## 2. Materials and Methods

### 2.1. Ethical Statement

This retrospective study adhered to the tenets of the Declaration of Helsinki and received ethical approval from the Institutional Review Board of Yeungnam University College of Medicine (IRB No. 2025-09-034). Due to the retrospective nature of the study, the requirement for obtaining informed consent was waived by the Institutional Review Board of Yeungnam University College of Medicine.

### 2.2. Study Design and Image Dataset

This retrospective imaging study included patients with pachychoroid spectrum disease who underwent UWF-ICGA examination at Yeungnam University Hospital between January 2022 and July 2025. The study was conducted in accordance with the tenets of the Declaration of Helsinki and was approved by the Institutional Review Board of Yeungnam University Hospital. The requirement for informed consent was waived because of the retrospective nature of the study. A total of 183 UWF-ICGA images from 183 eyes of 183 patients with pachychoroid spectrum disease were included. The mean age of the patients was 62.3 ± 9.2 years, and 97 patients (53.0%) were male and 86 (47.0%) were female. The diagnoses included central serous chorioretinopathy (CSC) in 65 patients (35.5%), pachychoroid neovasculopathy (PNV) in 62 (33.9%), and polypoidal choroidal vasculopathy (PCV) in 56 (30.6%). UWF-ICGA images were obtained using Optos California (Optos, Dunfermline, Scotland, UK). UWF-ICGA images were obtained during the early (2–3 min), middle (5–10 min), and late (10–15 min) phases after indocyanine green injection. Middle-phase images obtained 5–10 min after injection were used for IVA analysis, as the choroidal venous anatomy and vortex vein drainage patterns were clearly visualized during this phase. Images with insufficient quality for evaluation of the choroidal venous system were excluded.

### 2.3. Image Preprocessing

To reduce variation in image intensity across the dataset, brightness normalization was applied to the original UWF-ICGA images. For the object detection model, each original image was resized to 512 × 512 pixels using linear interpolation before model input. After automated quadrant detection, the identified quadrant regions were extracted as separate images. Because the extracted regions differed in their original dimensions, all quadrant images were resized to 512 × 512 pixels using linear interpolation before classification.

### 2.4. Definition and Labeling of Intervortex Venous Anastomosis

**Figure 1:**
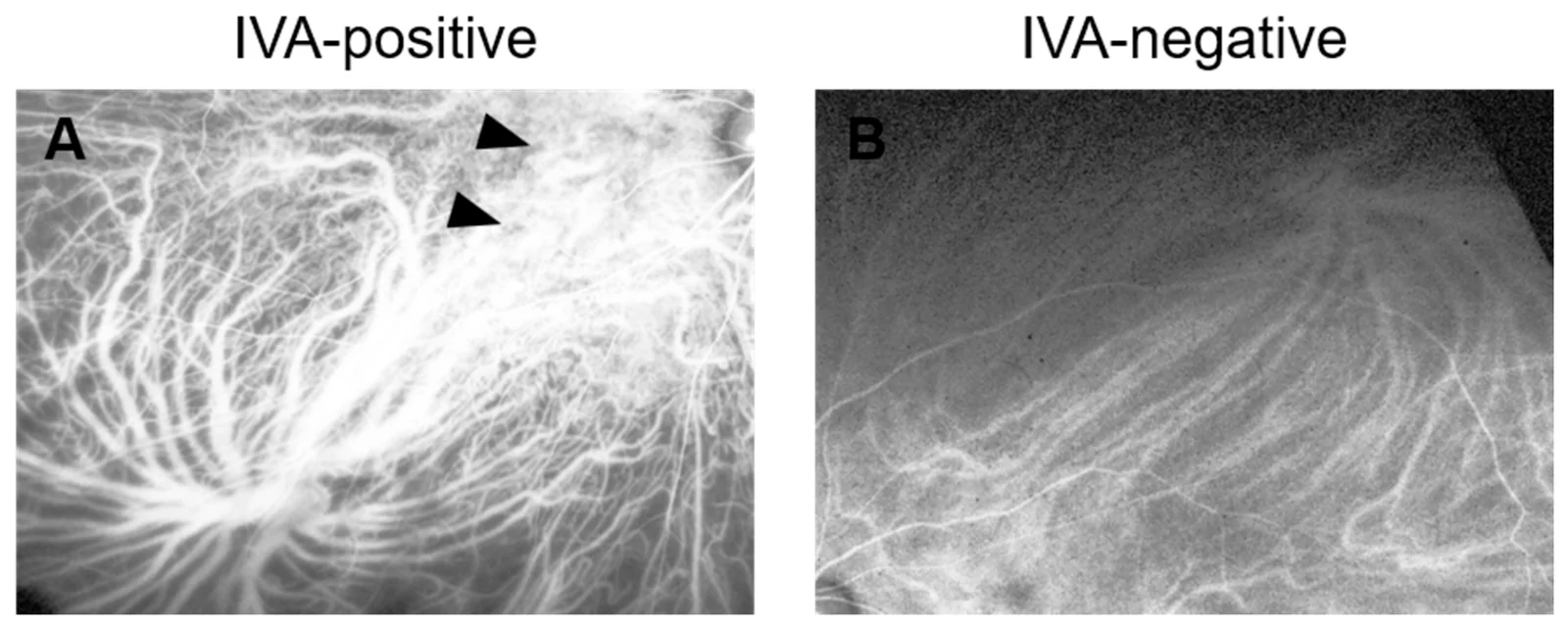
Representative UWF-ICGA images used for intervortex venous anastomosis classification. (**A**) Representative IVA-positive quadrant demonstrating a definite venous connection between adjacent vortex vein drainage territories (arrows). (**B**) Representative IVA-negative quadrant without an identifiable intervortex venous connection. During model development, IVA-positive and IVA-negative images corresponded to the internal labels “OK” and “NG”, respectively.

Each extracted quadrant was classified according to the presence or absence of IVA. IVA was defined as a clearly traceable venous channel crossing the presumed watershed boundary and establishing continuous communication between adjacent vortex vein drainage territories. Vessels that merely approached or overlapped the watershed region without demonstrable continuity between adjacent drainage territories were not considered IVA. When vascular continuity could not be confidently distinguished from the normal choroidal vascular network, the quadrant was classified as IVA-negative. During model development, IVA-positive and IVA-negative images corresponded to the internal labels OK and NG, respectively. Two retinal specialists (A.J. and M.S.) independently classified each quadrant while masked to the model predictions. Disagreements were resolved by consensus. The consensus classification served as the reference standard. Intergrader agreement was evaluated using Cohen’s kappa. The original performance report describes OK as a region in which the vessels were connected and NG as a region in which vascular continuity was not identified. Representative IVA-positive and IVA-negative quadrants are shown in Figure 1.

### 2.5. Deep Learning Framework

A sequential two-stage deep learning framework was developed consisting of:

Automated quadrant localization using an object detection model, followed by quadrant-level detection of IVA using a binary classification model.

**Figure 2:**
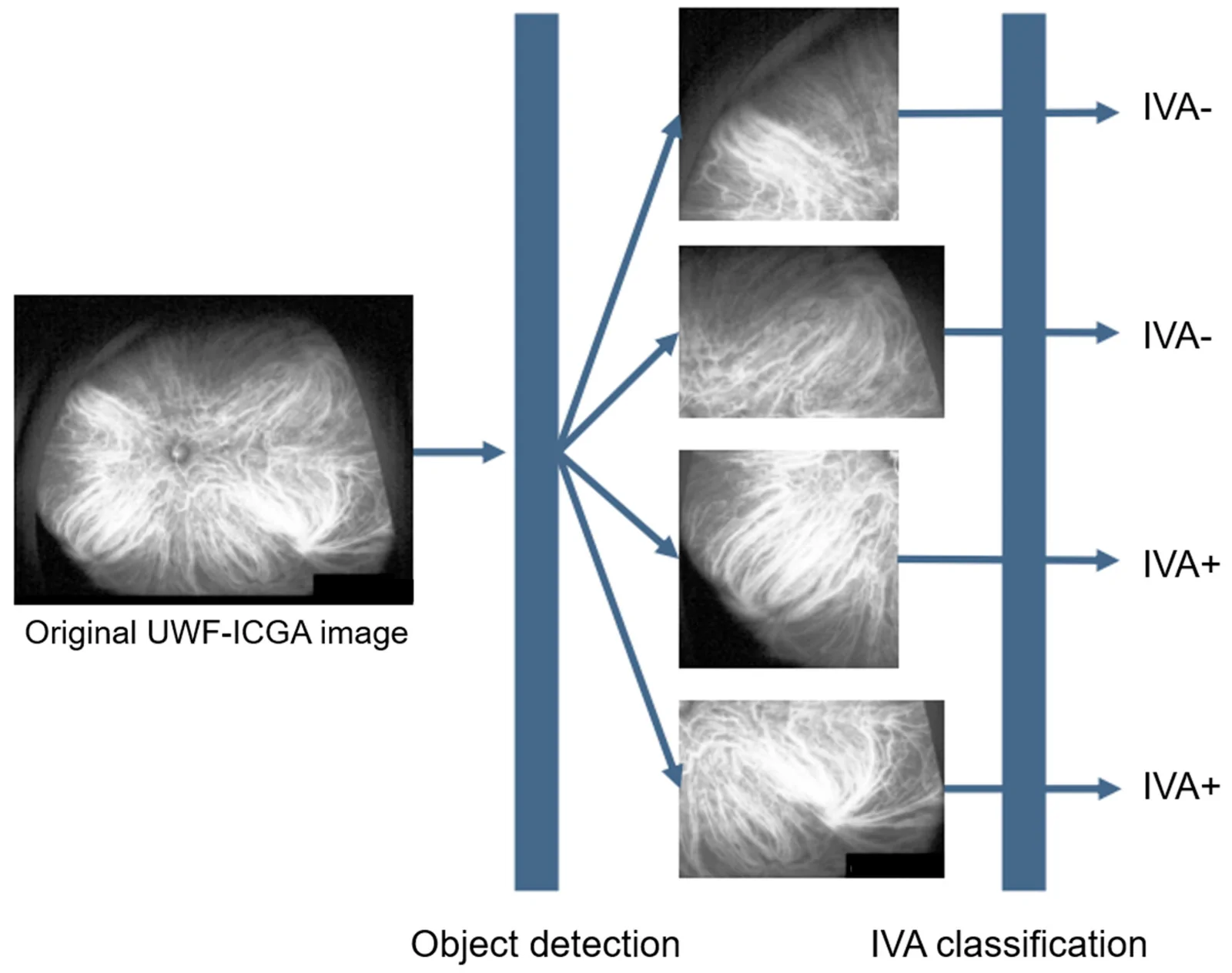
Overview of the two-stage deep learning framework for automated detection of intervortex venous anastomosis. The original ultra-widefield indocyanine green angiography (UWF-ICGA) image is first processed by an object detection model to automatically localize four predefined regions. Each detected region is subsequently extracted and analyzed independently by the classification model. Quadrants demonstrating definite intervortex venous anastomosis (IVA) are classified as IVA-positive, whereas those without identifiable anastomotic connections are classified as IVA-negative.

The complete dataset consisted of 183 original images and 732 corresponding quadrant images (183 × 4). The overall workflow of the proposed two-stage framework is illustrated in Figure 2.

Stage 1: Automated quadrant localization

The first stage consisted of an object detection model designed to identify the four predefined quadrants within each UWF-ICGA image. The object detection model, designated EYE4_OBD_QN, was developed to automatically identify four predefined regions of each UWF-ICGA image. A total of 183 images were used for model development. Of these, 155 images were included in the training dataset, and 28 independent images were used as the test dataset. The model received brightness-normalized 512 × 512-pixel images and generated four bounding-box coordinates for each image. The object detection model consisted of six layers and was trained for 318 epochs with a batch size of 28 using the Adam optimizer. The initial learning rate was 0.00146. Data augmentation was applied during model training. The software performance report indicates that the object detection model was trained using the Quick Learning (Normal) method.

Stage 2: Quadrant-level vascular pattern classification

The four quadrant images extracted from each original UWF-ICGA image were subsequently used as input for a binary classification model designated EYE_AD3. This resulted in a total of 732 quadrant images (183 images × four quadrants). These quadrant images were randomly divided at the quadrant level into 622 training images and 110 test images. The training dataset contained 362 IVA-negative and 260 IVA-positive quadrants. The test dataset contained 69 IVA-negative and 41 IVA-positive quadrants. The classification model consisted of 88 layers and was trained for 294 epochs with a batch size of 56 using the Adam optimizer. The initial learning rate was 0.007436. The classification model was trained using the Auto Deep Learning Level 3 setting, with data augmentation enabled. Detailed model configurations are summarized in Table 1.

### 2.6. Deep Learning Environment

All deep learning models were developed using Neuro-T version 4.2.0 (Neurocle Inc., Seoul, Republic of Korea), a no-code automated deep learning platform. Model training and evaluation were performed in an on-premises computing environment equipped with two NVIDIA Quadro RTX 5000 (NVIDIA Corporation, Santa Clara, CA, USA) graphics processing units with 16 GB memory, dual Intel Xeon processors, and 256 GB RAM.

### 2.7. Model Performance Evaluation

Performance of both deep learning models was evaluated using accuracy, precision, recall, and F1 score. For object detection, the number of labeled, predicted, and successfully matched bounding boxes was additionally evaluated. For IVA classification, model predictions were compared with reference labels using a confusion matrix. In addition to the software-generated performance metrics, class-specific sensitivity and specificity were calculated by considering IVA-positive as the positive class. Sensitivity was defined as the proportion of IVA-positive quadrants correctly classified as positive, whereas specificity was defined as the proportion of IVA-negative quadrants correctly classified as negative.

## 3. Results

### 3.1. Dataset Distribution

A total of 183 UWF-ICGA images were included in the study. For automated quadrant localization, 155 images were used for training and 28 images for testing. Each image was divided into four predefined regions, generating a total of 732 quadrant images for IVA classification. Of these, 622 were used for training and 110 for testing. The two retinal specialists demonstrated almost perfect agreement for quadrant-level IVA classification (Cohen’s κ = 0.84). Disagreements were resolved by consensus to establish the final reference labels. The training dataset consisted of 362 IVA-negative and 260 IVA-positive quadrants, whereas the test dataset consisted of 69 IVA-negative and 41 IVA-positive quadrants (Table 2).

### 3.2. Performance of the Object Detection Model

The object detection model was evaluated using 28 UWF-ICGA images. All labeled quadrants were correctly identified, with 112 predicted bounding boxes corresponding to 112 reference bounding boxes. The model achieved an accuracy of 100%, precision of 100%, recall of 100%, and F1 score of 100% (Table 3). All 28 test images were categorized as successfully detected without intermediate or failed detections.

### 3.3. Performance of the IVA Classification Model

**Figure 3:**
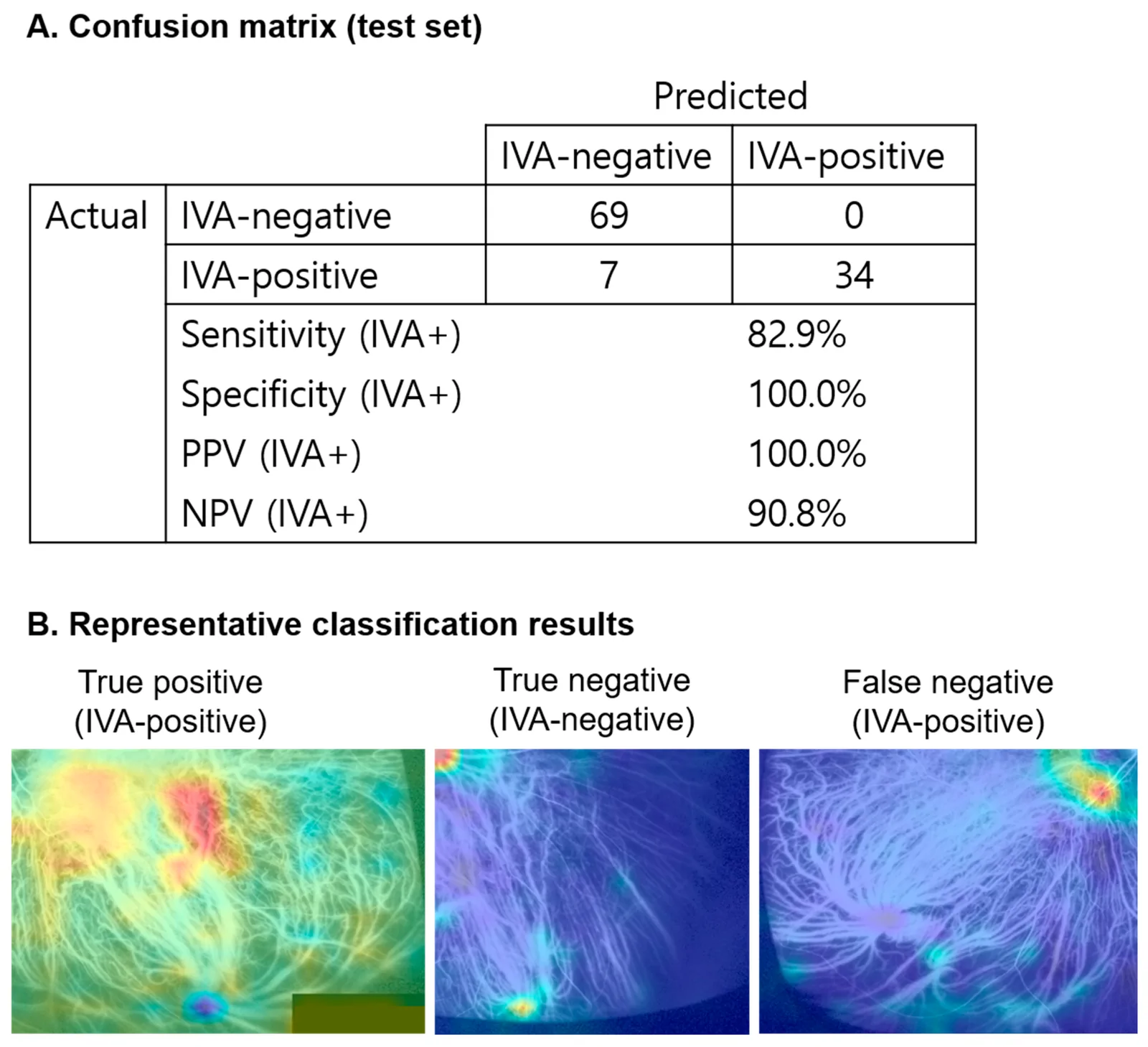
Performance of the deep learning model for quadrant-level detection of intervortex venous anastomosis (IVA). (**A**) Confusion matrix of the test dataset. All 69 IVA-negative quadrants were correctly classified, whereas 34 of 41 IVA-positive quadrants were correctly identified and seven were misclassified as IVA-negative. No false-positive IVA classification occurred. (**B**) Representative examples of correct and incorrect IVA classifications with activation heatmaps. Warmer colors (red and yellow) indicate regions with greater contribution to the model’s classification, whereas cooler colors (green and blue) indicate regions with lower contribution.

The classification model was evaluated using 110 quadrant images. The quadrant-level IVA classification model achieved an overall accuracy of 93.63%, precision of 95.39%, recall of 91.46%, and F1 score of 93.38%, according to the software-generated performance metrics (Table 3). Among the 69 IVA-negative quadrants in the test dataset, all 69 were correctly classified as IVA-negative. Among the 41 IVA-positive quadrants, 34 were correctly classified as IVA-positive, whereas seven were incorrectly classified as IVA-negative. No IVA-negative quadrant was incorrectly classified as IVA-positive. When IVA-positive was considered the positive class, the model therefore demonstrated a sensitivity of 82.9% (34/41) and specificity of 100% (69/69). The corresponding positive predictive value was 100% (34/34), whereas the negative predictive value was 90.8% (69/76). Thus, all classification errors in the test dataset represented false-negative detection of IVA, whereas no false-positive IVA detection occurred (Figure 3).

## 4. Discussion

In this study, we developed a two-stage deep learning framework for automated detection of intervortex venous anastomosis (IVA) on UWF-ICGA. The proposed system first localized predefined regions of each UWF-ICGA image and subsequently classified each region according to the presence or absence of IVA. The object detection model demonstrated perfect localization performance in the internal test dataset, while the IVA classification model achieved an overall accuracy of 93.63%. These findings demonstrate the feasibility of automated regional assessment of intervortex venous connections on UWF-ICGA.

IVA has attracted increasing attention as an imaging manifestation of choroidal venous remodeling. Matsumoto et al. identified superior-inferior vortex vein anastomoses in more than 90% of eyes with central serous chorioretinopathy (CSC), pachychoroid neovasculopathy (PNV), or polypoidal choroidal vasculopathy (PCV), substantially more frequently than in healthy eyes [2,7]. Spaide et al. further showed that IVA distribution varies across pachychoroid-related disorders, with connections predominantly centered in the macula in CSC and distributed more extensively around the optic disc in peripapillary pachychoroid syndrome [4]. Fernández-Vigo et al. similarly reported prominent macular anastomoses more frequently in eyes with chronic CSC than in unaffected fellow eyes and healthy controls [5]. These observations indicate that both the presence and spatial distribution of IVA may reflect disease-related reorganization of choroidal venous drainage. In particular, the peripapillary region may represent another relevant region of interest because of its close relationship with the horizontal and vertical choroidal watershed zones. Future extensions of the present framework incorporating peripapillary, macular, and other region-specific analyses may therefore provide a more comprehensive characterization of disease-specific patterns of choroidal venous remodeling.

The potential importance of IVA is supported by the venous overload choroidopathy framework proposed by Spaide et al. [3]. According to this model, impaired choroidal venous outflow increases venous pressure and promotes dilation or recruitment of pre-existing collateral channels between adjacent vortex vein systems. Experimental vortex vein occlusion has produced pachychoroid-like changes and remodeling of choroidal drainage routes, supporting a compensatory role for these connections [8]. In pachychoroid spectrum diseases, dilated IVA and adjacent pachyvessels have also been reported to colocalize with areas of choroidal vascular hyperpermeability, linking structural venous remodeling with choroidal congestion and leakage [3,7]. Choroidal vascular hyperpermeability has, in turn, been associated with anatomical response to anti-vascular endothelial growth factor therapy in pachychoroid-related neovascular diseases [9,10,11,12,13,14]. These findings raise the possibility that automated IVA assessment could contribute to future studies of disease activity and treatment response.

A major challenge in IVA research is the lack of a universally accepted definition. Matsumoto et al. [2] emphasized large vessels connecting superior and inferior vortex vein systems without narrowing toward the watershed zone, whereas Fernández-Vigo et al. [5] applied a diameter threshold of 150 μm to macular connections crossing the temporal watershed zone. By contrast, our reference labels were based on the qualitative identification of definite vascular continuity by two retinal specialists, without prespecified thresholds for vessel number or caliber. The high Cohen’s kappa indicates that this operational definition was reproducible between the two graders.

The quadrant-based architecture was selected to reduce the spatial complexity of the ultra-widefield choroidal venous network. Conventional macula-centered imaging may incompletely represent nasal and peripheral venous connections, whereas UWF-ICGA can visualize multiple vortex vein drainage territories and their peripheral portions within a single image [3,6]. Direct classification of the entire UWF-ICGA image could allow small regional connections to be obscured by extensive background vasculature. By first extracting standardized regions and then evaluating each region separately, the proposed framework preserves spatial information while simplifying the classification task. This approach provides information complementary to global vascular metrics such as choroidal vessel density, which quantify overall vascular abundance but may not fully capture abnormal connectivity between distinct vortex vein drainage territories. Notably, a recent study introduced the Index of Vortex Anastomoses Number (IVAN), defined as the number of peripapillary quadrants showing vascular connectivity, and demonstrated its association with clinical characteristics of CSC [15]. This finding supports the potential value of quadrant-based assessment as a semiquantitative measure of IVA burden. Similarly, our automated quadrant-level framework could provide the basis for an eye-level IVA score based on the number and distribution of IVA-positive quadrants, facilitating future investigation of associations with disease phenotype, severity, and treatment response.

The confusion matrix provides additional insight into the behavior of the classification model. All 69 IVA-negative quadrants were correctly classified, whereas seven of 41 IVA-positive quadrants were missed. Thus, positive predictions were highly reliable within the present test dataset, but a negative model output could not exclude IVA. This error pattern suggests that the classifier favored specificity over sensitivity. Subtle vascular continuity, incomplete visualization of an anastomotic vessel within an extracted region, or reduced image quality in the peripheral field could plausibly contribute to false-negative predictions. However, because the seven false-negative cases were not formally characterized, these explanations remain hypothetical.

Automated analysis may be particularly useful for IVA because manual evaluation requires careful inspection of extensive and complex vascular networks and may be affected by image quality and observer experience. One study demonstrated the difficulty of integrating widefield choroidal vascular information and suggested that automation could facilitate assessment of intervortex connections [3]. The present model represents an initial step toward this goal by converting expert-based regional assessment into a reproducible computational task.

Several limitations should be acknowledged. The dataset was relatively small and derived from a single-center image cohort, and external validation was not performed. Moreover, binary quadrant-level classification does not provide information regarding vessel segmentation, caliber, extent, or flow characteristics. Patient-level clinical and imaging variables were not analyzed, precluding assessment of relationships between automatically detected IVA and pachychoroid phenotype, choroidal hyperpermeability, disease activity, or treatment response. An additional limitation concerns the data-splitting strategy for the Stage 2 classification model. The training and test sets were randomly divided at the quadrant level rather than at the patient or eye level. Consequently, quadrants originating from the same eye could have been included in both datasets, potentially introducing anatomical similarity between the training and test sets and leading to an optimistic estimate of classification performance. Future studies should employ strictly patient-level data separation and external validation in independent datasets to confirm the generalizability of the model.

In conclusion, we developed a two-stage deep learning system for automated detection of IVA on UWF-ICGA. By enabling objective identification of a key feature of choroidal venous remodeling, this approach may provide a useful imaging tool for future studies of venous overload choroidopathy and pachychoroid spectrum diseases.

## Figures and Tables

**Table 1:** Configuration of the two-stage deep learning framework.

Parameter	Object Detection	IVA Classification
Model	EYE4_OBD_QN	EYE_AD3
Purpose	Quadrant localization	IVA classification
Input	UWF-ICGA	Extracted quadrant
Input size	512 × 512 px	512 × 512 px
Preprocessing	Brightness normalization, resizing	Resizing
No. of layers	6	88
Batch size	28	56
Epochs	318	294
Optimizer	Adam	Adam
Initial learning rate	0.00146	0.007436
Software	Neuro-T v4.2.0	Neuro-T v4.2.0

**Table 2:** Dataset composition for development and evaluation of the deep learning models.

Dataset	Total	Training	Test
Original UWF-ICGA images	183	155	28
Extracted quadrant images	732	622	110
IVA-negative quadrants	431	362	69
IVA-positive quadrants	301	260	41

**Table 3:** Performance of the two-stage deep learning framework in the test datasets.

Performance Metric	Quadrant Localization	IVA Classification
Accuracy	100.0%	93.63%
Precision *	100.0%	95.39%
Recall *	100.0%	91.46%
F1 score *	100.0%	93.38%
Sensitivity for IVA	—	82.9%
Specificity for IVA	—	100.0%
PPV for IVA	—	100.0%
NPV for IVA	—	90.8%

* Accuracy, precision, recall, and F1 score represent software-generated performance metrics. Sensitivity, specificity, positive predictive value (PPV), and negative predictive value (NPV) were additionally calculated from the confusion matrix with IVA-positive considered the positive class.

## Data Availability

The original contributions presented in this study are included in the article. Further inquiries can be directed to the corresponding author.
